# The Independent and Shared Mechanisms of Intrinsic Brain Dynamics: Insights From Bistable Perception

**DOI:** 10.3389/fpsyg.2018.00589

**Published:** 2018-04-24

**Authors:** Teng Cao, Lan Wang, Zhouyuan Sun, Stephen A. Engel, Sheng He

**Affiliations:** ^1^State Key Laboratory of Brain and Cognitive Science, Institute of Biophysics, Chinese Academy of Sciences, Beijing, China; ^2^College of Life Sciences, University of Chinese Academy of Sciences, Beijing, China; ^3^Department of Psychology, University of Minnesota, Minneapolis, MN, United States

**Keywords:** bistable perception, perceptual rivalry, temporal dynamics, correlation matrix, clustering

## Abstract

In bistable perception, constant input leads to alternating perception. The dynamics of the changing perception reflects the intrinsic dynamic properties of the “unconscious inferential” process in the brain. Under the same condition, individuals differ in how fast they experience the perceptual alternation. In this study, testing many forms of bistable perception in a large number of observers, we investigated the key question of whether there is a general and common mechanism or multiple and independent mechanisms that control the dynamics of the inferential brain. Bistable phenomena tested include binocular rivalry, vase-face, Necker cube, moving plaid, motion induced blindness, biological motion, spinning dancer, rotating cylinder, Lissajous-figure, rolling wheel, and translating diamond. Switching dynamics for each bistable percept was measured in 100 observers. Results show that the switching rates of subsets of bistable percept are highly correlated. The clustering of dynamic properties of some bistable phenomena but not an overall general control of switching dynamics implies that the brain’s inferential processes are both shared and independent – faster in constructing 3D structure from motion does not mean faster in integrating components into an objects.

## Introduction

Our visual brain constantly engages in the inferential process of constructing a meaningful and coherent interpretation of the visual world based on retinal images, a process mostly unconscious to the observers (Helmholtz, 1962). However, some people may be faster than others in making these “inferences.” Is there a general and common mechanism or multiple and independent mechanisms that control the dynamics of the inferential brain? Bistable perception, where viewing a constant and stable stimulus leads to the dynamic alternation between two interpretations (Sterzer et al., 2009), provides an opportunity to test the common vs. independent nature of brain’s dynamics.

There are many forms of bistable perception, well-known examples include binocular rivalry (competition between different images from the two eyes), alternating face or vase perception, switching direction of motion with structure-from-motion displays, etc. (Leopold and Logothetis, 1999; Blake and Logothetis, 2002; Pearson and Brascamp, 2008). A key property of bistable perception is the spontaneous nature of the perceptual alternations, the dynamics of which presumably reflects the dynamics of our brain’s inferential process. The many different forms bistable perception share similar dynamic properties (Brascamp et al., 2005; van Ee, 2005; Sheppard and Pettigrew, 2006). Perceptual alternation is typically stochastic, with the probability distribution of dominance time following a gamma distribution (Lehky, 1995). In the case of binocular rivalry, the dominance durations are only minimally affected by voluntary control (Meng and Tong, 2004; Chong et al., 2005; Klink et al., 2008), yet attention is critical for its manifestation (Chong and Blake, 2006; Kohler et al., 2008; Dieter and Tadin, 2011; Zhang et al., 2011; Brascamp and Blake, 2012).

Though the alternation dynamics of different bistable perception share common statistical properties, the specific switching rates of different stimuli can have large differences. More importantly, there are large individual differences in switching rates for bistable phenomena (Carter and Pettigrew, 2003; Patel et al., 2015). The individual differences in switching rates present an opportunity to investigate the critical question about whether there is a general mechanism or multiple independent mechanisms supporting the inferential processes that underlie the dynamics of the inferential brain?

One scenario is that there is a common temporal mechanism such as a master clock that is responsible for the timing of all dynamic switching processes. For example, the idea that in bistable perception, the switching occurs between representations in the two hemispeheres (Miller et al., 2000). However, there is also evidence that suggests different cortical areas are involved in switching dynamics (Kanai et al., 2005), such as the interocular completion between monocular neurons in V1 for binocular rivalry (Blake and Logothetis, 2002); the activity of prefrontal cortex (Windmann et al., 2006), inferior frontal cortex (Sterzer and Kleinschmidt, 2007), and frontoparietal cortex (Lumer et al., 1998; Knapen et al., 2011; Weilnhammer et al., 2013) might influence the rivalry rate. Another evidence from morphology supported that local gray-matter density in the parietal cortex might influence the dominance time (Kanai et al., 2010, 2011). However, even if different bistable perception may transpire at different cortical sites, the neuronal dynamics underlying the switching could still have shared properties.

An important alternative scenario is that different bistable phenomena are controlled by their respective cortical mechanisms with relatively independent dynamic properties. Indeed, many factors could potentially influence the switching rates, including local level of neural noise, dynamic properties of adaptation, etc. (van Ee, 2009; Pastukhov and Braun, 2011; Scocchia et al., 2014). Even relatively global factors such as mood of observer at that time (Sheppard and Pettigrew, 2006), while it is possible that different processes are differentially influenced by those global factors.

The following general factors might influence the dynamics of alternation: gender (Schouten et al., 2010), age (Beer et al., 1989), visual acuity, color vision, basic stereovision, handedness (Christman et al., 2009), simple reaction time (Schouten et al., 2013), and anxiety (Nagamine et al., 2007). If some of these factors have connection to the alternation rate, it would be helpful to understand the fundament of percept switch.

In the present study, we use an individual difference approach to investigate whether there is common mechanism that either control or influence the dynamics of the visual brain, by examining the correlation among the switching rates between different bistable perceptions across individuals. In other words, if an observer experiences faster switching in binocular rivalry, will the same observer also experience faster switching in (some) other bistable perception?

## Materials and Methods

### Main Experiment

#### Stimuli and Apparatus

**FIGURE 1:**
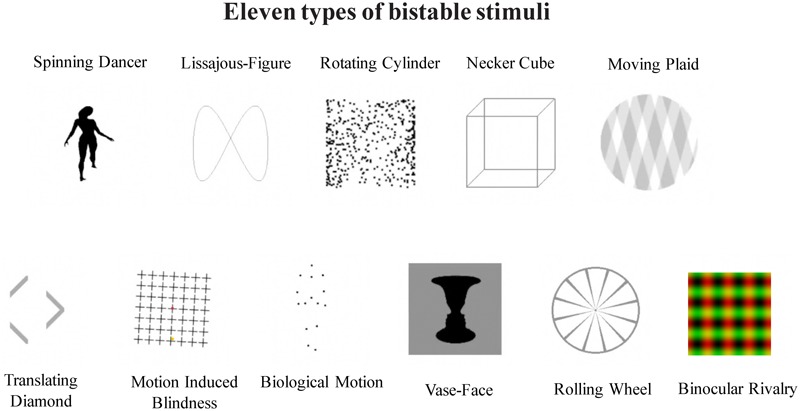
Illustration of 11 different types of bistable stimuli, as labeled. Top panel: Biological Motion (BM), Vase-Face (VF), Translating Diamond (TD), Lissajous-figure (LF), and Motion induced Blindness (MB); bottom panel: Moving Plaid (MP), Necker Cube (NC), Rotating Cylinder (RC), Rolling Wheel (RW), and Spinning Dancer (SD).

A total of 11 bistable stimuli were used in the main experiment, including 10 types of monocular bistable stimuli, and binocular rivalry (**Figure 1**).

For binocular rivalry, horizontal red/black and vertical green/black gratings were dichoptically presented through a stereoscope to observers. The gratings were 0.775 cpd sinewave gratings extending 3.1 × 3.1°, centered at the fixation point.

The other stimuli were observed binocularly without stereoscope, and all were similar in sizes (∼3°). The vase-face and motion induced blindness stimuli were also presented at the fixation point, however, the other 8 types of stimuli were presented 2.2° below the fixation point, to minimize the potential of stimulus-induced or voluntary eye movements that might influence the switch dynamics. The fixation point was a 0.13° red dot.

Subjects were seated at a distance of 57 cm from a 19-inch CRT monitor, with a resolution of 1280 × 960 and a refresh rate of 90 Hz, and their head was stabilized using a chin and forehead rest.

For static stimuli (VF, NC, BR), the figures were continuously presented (**Figure 1**). Below, we provide brief description for each type of the dynamic stimuli.

(1) SD: Stimuli were obtained from Liu et al. (2012), figure was manipulated in length and width, the color was reversed, and each gait cycle was 1.13 s and contained 102 frames.
(2) LF: Lissajous-figure was generated by the intersection of two sinusoids with perpendicular axes [x(*t*) = sin(2*t*); y(*t*) = sin(*t* + ∂); with ∂ increasing from 0 to 2 pi], and rotated 0.25 cycle per second (Weilnhammer et al., 2014).
(3) RC: Cylinder projections were 3.1° wide and 3.1° tall, contained 450 randomly placed dots, and rotated 0.25 cycles per second.
(4) MP: The gratings were 0.75 cpd rectangular wave gratings extending 3° of visual angle in diameter. The gratings moved at a speed of 1.5°/s.
(5) TD: Stimuli was a line drawing of a diamond whose four corners were occluded by three vertical bars of the same color as the background, the diamond moved at a constant horizontal speed of 3°/s and reversed direction every 1 s (Fang et al., 2008).
(6) MIB: Stimuli consisted of a yellow dot and a red fixation dot, overlaid on a global moving pattern of 49 blue crosses, which was moving clockwise 0.25 cycle per second. The yellow dots subtended 0.2° of visual angle arranged 1.4° of visual angle below the fixation dot.
(7) BM: Point-light human was adopted from (Shi et al., 2010), the head and joint positions in each frame were encoded as motion vectors with initial starting positions, and each gait cycle was 0.94 s and contained 85 frames.
(8) RW: Stimuli had 12 spokes rotating at 0.375 cycle per second. It was a type of apparent motion.

#### Procedure and Task

At the beginning of the experimental session, each subject went through a training process in which the subject was first shown the two possible percepts of the stimuli, then experienced the alternating perception 16 times (with button presses). After training, the task session began with 2 binocular rivalry trials, followed by the other 10 bistable stimuli in a random order with each type repeated three times, then binocular rivalry was tested twice again.

Each trial started with a press of the space key by the subject, and the stimuli were presented for 60 s. During that period, subjects pressed one of two keys indicating their dominant percepts. Subjects were informed to maintain their fixation throughout each trail. All had 60-s rest between trials.

#### Participants

A total of 100 subjects (53 females) with normal or corrected to normal vision participated, their age ranged from 18 to 33, with the majority between 20 and 25. Prior to participating in the bistable experiment, they were tested in: visual acuity, stereo acuity, color vision, simple reaction time, perception of coherent motion, and handedness.

#### Data Analysis

For each subject and each stimulus type, we obtained the average dominance time from the three 60-s trials (four trials for binocular rivalry); then the average switch rates were also calculated. For each type the switching rate data of observers was eliminated which exceeding 3 standard deviations among 100 observers. In total 9 data points from 1100 were excluded in correlation analysis.

The MATLAB and SPSS were used to analyze the data. To investigate the similarity of each bistable stimuli, correlation coefficient was calculated between each stimuli type pair. We applied factor analysis to see whether there are some latent factors correspond for the similarity pattern. In order to view how dissimilarities contribute the bistable stimuli relationship, multi-dimensional scaling was done in 3D, because the initial eigenvalues of first three components exceed 1, and for better individual distributions, we used standardized logarithmic switch rates.

### Control Experiments

We performed two control experiments in smaller number of subjects to investigate (1) whether the variation of switching dynamics across individuals was largely invariant to the retinal location tested; and (2) the potential contribution of eye blinking and movement patterns into the switch dynamics.

**FIGURE 2:**
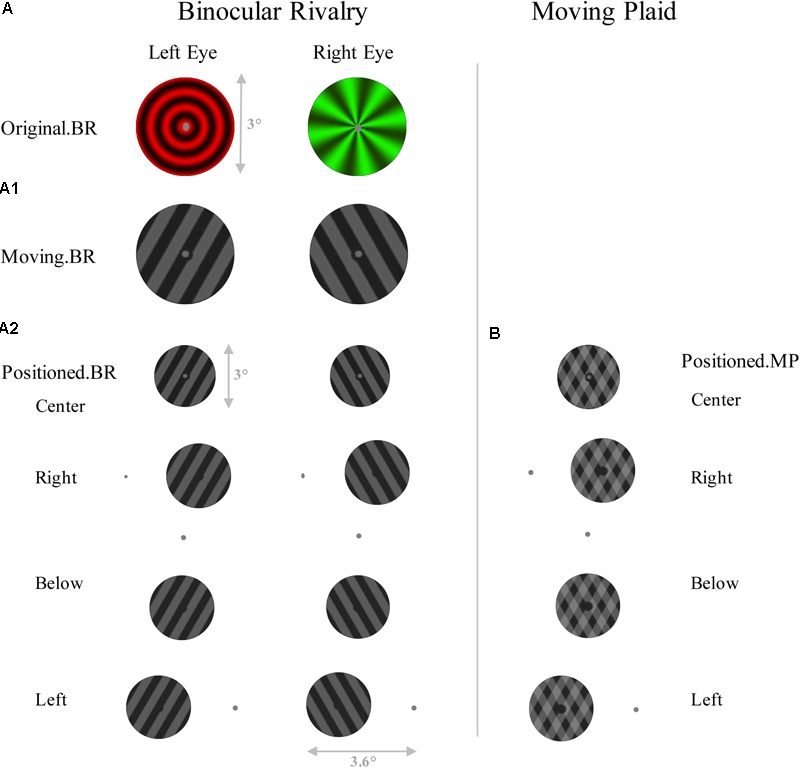
Schematic representation of the stimuli of control experiment 1. Left panel shows six conditions of Binocular rivalry, including **(A)** original binocular rivalry pattern, **(A1)** a 60° angled set of gratings which are moving in opposite directions, and **(A2)** gratings which are placed in different positions to the fixation. **(B)** Right panel shows four conditions of Moving plaid, which have the same schema as **A2**.

In the first control experiment, with 13 subjects (8 females), the procedures were the same as described above, except that only the binocular rivalry and moving plaid stimuli were used, and presented at different location to the fixation with pattern changes as **Figure 2**. The fixation dot was gray and had a diameter of 0.2°, and the hole in the center was 0.4°. Physical presentation condition was the same as the main experiment. And all viewed through stereoscope.

In the second control experiment (*n* = 14, 9 female) aimed at investigating the contribution of eye movements. Visual stimuli (rolling wheel, vase-face, binocular rivalry, Necker cube, rotating cylinder, moving plaid, and translating diamond) were presented 5 × 5° on a 21-inch monitor, with a resolution of 1920 × 1440 and a refresh rate of 75 Hz.

The procedure and task were essentially the same as the main experiment, except the addition of eye tracking. Eye movements and blinks were measured by using a noninvasive infrared “Eyelink-1000” (SR Research, Osgoode, Ottawa, ON, Canada) eye tracker with sample rate 500 Hz binocularly.

## Results

The main experiment investigated the relationship between temporal dynamics of different bistable stimuli, with an individual difference approach. A simple and direct question is whether some of the switching rates are correlated across subjects? The basic results are in the form of cross-individual correlations between different stimulus types.

### Correlation Matrix

**FIGURE 3:**
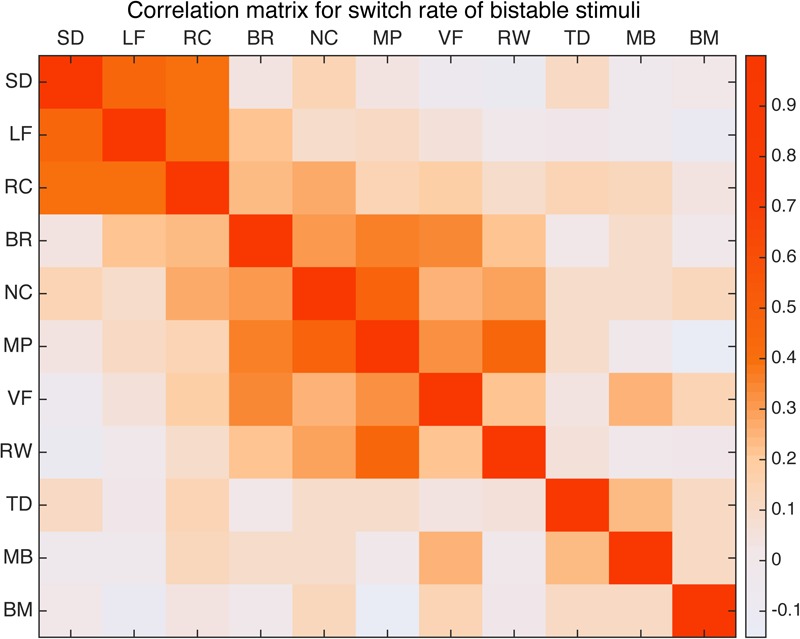
Correlation matrix for 11 bistable stimuli. Warm color indicates relative strong correlation. Spinning dancer (SD), Lissajous-figure (LF), rotating cylinder (RC), binocular rivalry (BR), Necker cube (NC), moving plaid (MP), vase-face (VF), rolling wheel (RW), translating diamond (TD), motion induced blindness (MB), and biological motion (BM).

First we computed the Pearson’s correlation coefficient between the switching rates of all 11 bistable types across our sample of 100 subjects. The paired correlations are then put together to form a correlation matrix, shown in **Figure 3** as a heatmap. To correct for multiple comparisons, significance is asserted only for correlation coefficient with a corrected *p*-value below that corresponding to an expected false discovery rate (FDR) of 0.05. The significant paired correlations are also summarized in **Table 1**.

**Table 1:** Significant correlation values of notable pair-wise correlations.

	SD – LF	MP – NC	MP – RW	SD – RC	LF – RC	BR – VF	MP – BR
Pearson *r*	0.463	0.462	0.403	0.393	0.371	0.369	0.344
*p*-value (corrected)	0.001	0.001	0.002	0.004	0.009	0.009	0.027

**FIGURE 4:**
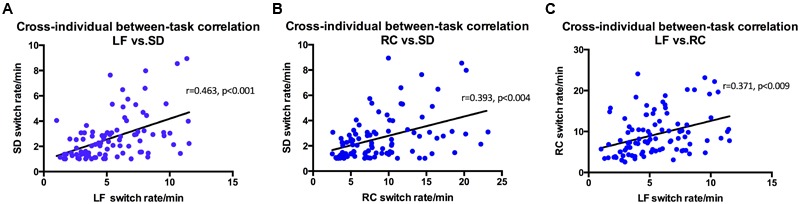
Switching rates correlations for Rotating Cylinder, Spinning Dancer, and Lissajous-figure. **(A)** Using intra-class correlational analysis, we found moderate-level concordance in switching rates between Lissajous-figure and Spinning Dancer (*r* = 0.463, *p* < 0.001). Similarly, **(B)** Rotating Cylinder and Spinning Dancer (*r* = 0.393, *p* < 0.004) and **(C)** Lissajous-figure and Rotating Cylinder (*r* = 0.371, *p* < 0.009).

Perhaps not that surprisingly, results show that the three stimuli related to structure from motion (spinning dancer, Lissajous-figure, and rotating cylinder) were highly correlated with each other (**Figure 4**). Correlation between SD and LF was *r* = 0.463 (*p* < 0.001), SD and RC was *r* = 0.393, (*p* < 0.004), and LF and RC was *r* = 0.371 (*p* < 0.009).

**FIGURE 5:**
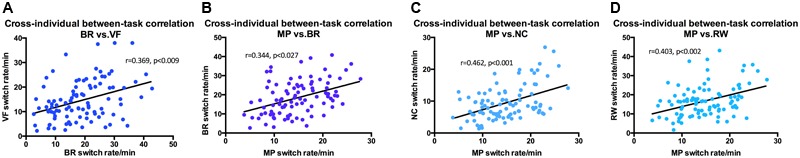
Switching rates correlations for Binocular Rivalry, Vase-face, Moving Plaid, Necker Cube, and Rolling Wheel. **(A)** Binocular Rivalry and Vase-Face (*r* = 0.369, *p* < 0.009). **(B)** Binocular Rivalry and Moving Plaid (*r* = 0.344, *p* < 0.027). **(C)** Moving Plaid and Necker Cube (*r* = 0.462, *p* < 0.001). **(D)** Moving Plaid and Rolling Wheel (*r* = 0.403, *p* < 0.002).

In addition, binocular rivalry was also correlated with a number of other stimuli (**Figure 5**), the strongest was with vase-face *r* = 0.369 (*p* < 0.009). Moving plaid had relative strong correlation with Necker cube *r* = 0.462 (*p* < 0.001), and with rolling wheel *r* = 0.403 (*p* < 0.002), as well as with binocular rivalry *r* = 0.344 (*p* < 0.027).

### Structures (Clustering) in the Correlation Matrix: Factor Analysis and Multi-Dimensional Scaling

In order to see whether there are some latent components that can explain the pattern in the correlation matrix, we performed factor analysis in 11 stimuli types across individuals. The Kaiser–Meyer–Olkin measure of sampling adequacy was 0.662. A minimum Kaiser–Meyer–Olkin score of 0.50 is considered necessary to reliably use factor analysis for data analysis. Similarly, the Bartlett test of sphericity (the higher the better) was 163.7 with significance level of *p* < 0.000. The inspection of the Scree plot and eigenvalues produced a departure from linearity coinciding with a three-factor result. Therefore, the Scree test indicated that the data should be analyzed for three factors. To facilitate interpretation of results, Orthogonal Varimax rotation was done. From **Table 2**, we could see different stimuli contribute to factors differently: Binocular Rivalry, Vase-face, Moving Plaid, Necker Cube, and Rolling Wheel contribute to the first factor most, Rotating Cylinder, Spinning Dancer, and Lissajous-figure loaded highly on the second factor, and the other three contribute to the third.

**Table 2:** Rotated component matrix of factor analysis.

Rotated component matrix			
	Component		
	1	2	3
SD	-0.085	0.81	0.016
LF	0.088	0.79	-0.147
RC	0.237	0.697	0.222
BR	0.626	0.193	0.039
NC	0.647	0.19	0.165
MP	0.811	0.058	-0.143
VF	0.576	-0.019	0.368
RW	0.679	-0.155	-0.088
TD	0.046	0.14	0.555
MB	0.074	-0.005	0.717
BM	-0.053	-0.089	0.613

*Extraction method: principal component analysis.*

**FIGURE 6:**
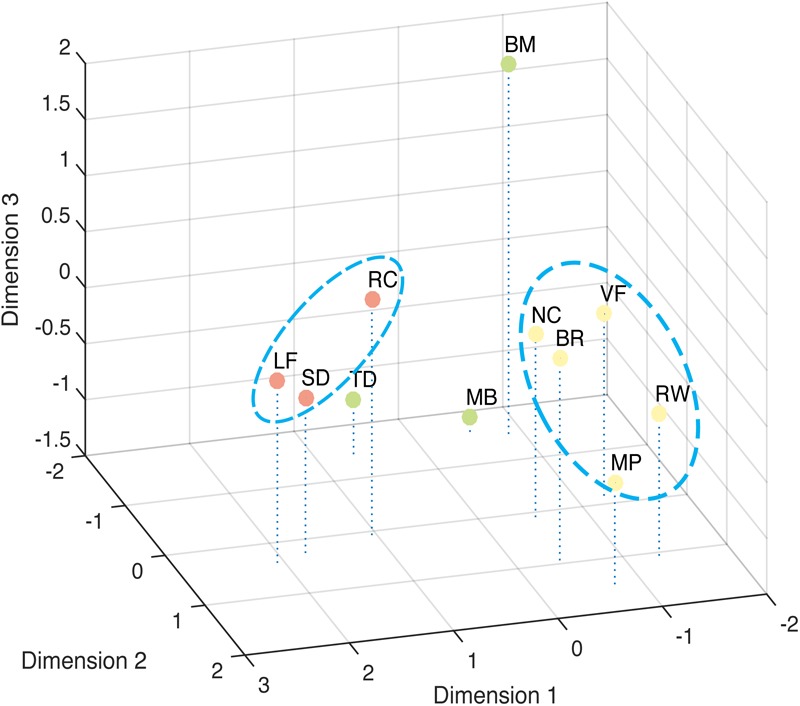
Multi-Dimensional Scaling of tested bistable types. Dissimilarity measured by Euclidean distance model. Closer distance indicates higher similarity.

The correlation result show to what extent the stimuli were lineally alike, and the multi-dimensional scaling could provide a more intuitive view of how these different stimuli are grouped (**Figure 6**). The best solution for the dissimilarity coefficient matrix for the 11 stimuli types at this data scale was computed by ALSCAL as a 3D Euclidean space: stress value 0.105, RSQ = 0.910. From the figure, we found the 11 stimuli clustered into configurations consistent with the results from factor analysis. As indicated in the figure, Rotating Cylinder, Spinning Dancer, and Lissajous-figure are close with each other, and vase-face, Necker cube, rolling wheel, binocular rivalry, and moving plaid seem to form another group.

### Influence of Gender and Age to Switching Dynamics

**FIGURE 7:**
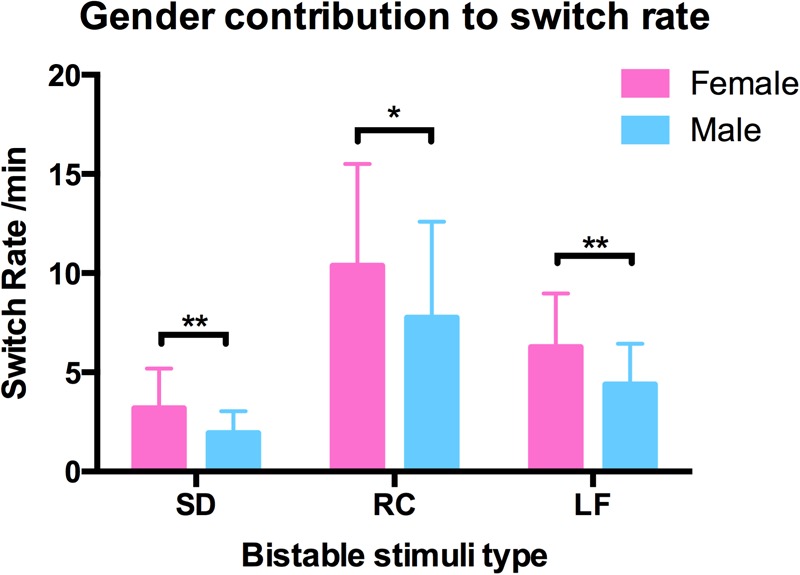
Gender contribution in switching rates in three bistable types spinning dancer (SD), rotating cylinder (RC), and Lissajous-figure (LF) for male (blue) and female (red) observers, and males had a slower switching rates than females in SD (^∗∗^*p* < 0.001), RC (^∗^*p* < 0.011), LF (^∗∗^*p* < 0.001).

**FIGURE 8:**
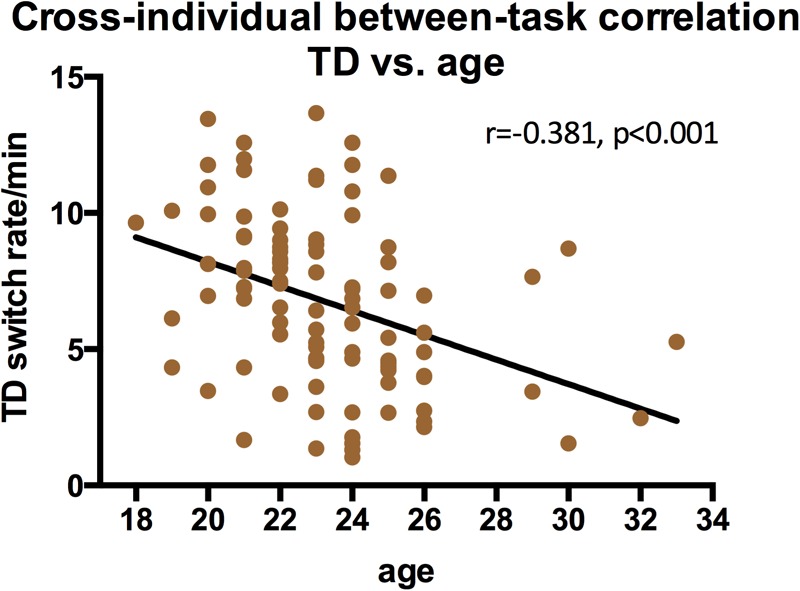
Switching rates correlations for translating diamond and age (*r* = –0.381, *p* < 0.001).

We also examined the contribution of gender and age to switching rates, and discovered that gender strongly influenced the switching dynamics of the three types of structure-from-motion stimuli (SD, RC, and LF, see **Figure 7**), that males had a significantly slower switching rates than females in spinning dancer (*F* = 14.25, *p* < 0.001), rotating cylinder (*F* = 6.80, *p* < 0.011), and in Lissajous-figure (*F* = 15.78, *p* < 0.001). In addition, there is also a negative correlation between switching rates of translating diamond and age, slower switching of translating diamond with increasing age (**Figure 8**).

### Control Experiments

The two control experiments were performed to investigate how sensitive the alternation dynamics were to the retinal location and eye movements.

**FIGURE 9:**
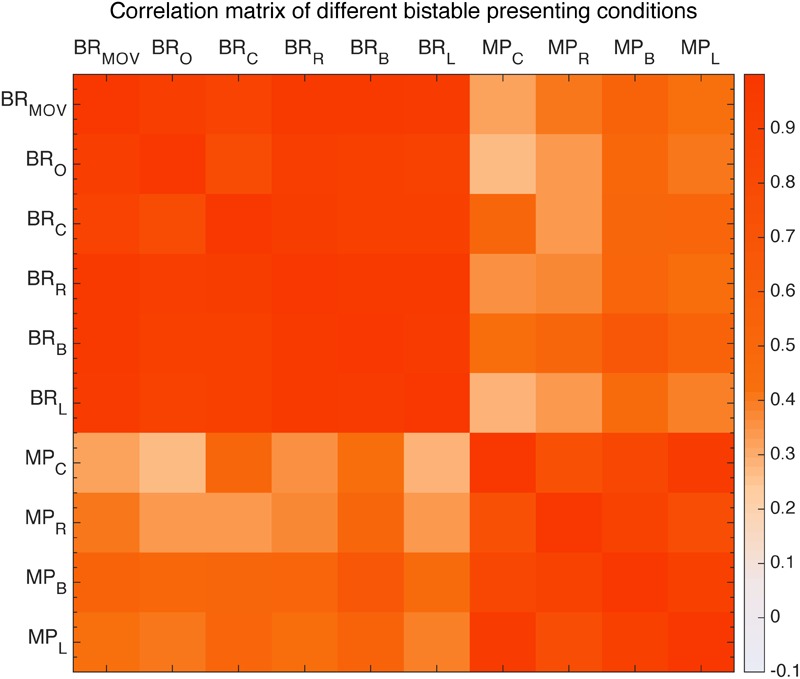
Correlation matrix of different presentation conditions of moving plaid and binocular rivalry. From the left: first six are binocular rivalry with moving pattern (BR_MOV) and original pattern (BR_O) and four different positions to fixation conditions: center (BR_C), right (BR_R), below (BR_B), left (BR_L), the other four are moving plaid presented at view center (MP_C), right (MP_R), below (MP_B), left (MP_L).

First, we looked at the effects of retinal location. With Binocular Rivalry stimuli and the Moving Plaids presented at different retinal locations (see the section “Materials and Methods”), their relative switching rates seem to be highly invariant. We plotted the Pearson’s correlations between tested conditions in a correlation matrix (**Figure 9**). The results clearly showed that the correlation was essentially invariant within the same stimulus type, invariant to the stimulus location (diagonal cells in the matrix, all *r* > 0.7 and *p* < 0.001), and a reduced level of correlation between the two stimulus types was also largely invariant to the stimulus location (the off-diagonal cells, *r*∼0.4).

Then, we examined the effect of eye moments and blinks in a small group of subjects. Eye movements and blinks of both eyes in 14 observers were recorded at 500 Hz during exposure to seven different types of rivalry stimuli. The number of blinks of every subject was calculated by the missing of pupil during constant presentation of every stimulus. Results show that there was no correlation between the number of blinks and the perception switching rates across individuals (**Table 3**, top row). The mean absolute Pearson’s correlation coefficients value is 0.140, and all *p* > 0.25. Data indicated that blink rates were relatively constant when viewing different stimuli, suggesting that variations in eye blink is very unlikely the cause of the observed clustering among the 11 stimuli in the main results.

**Table 3:** Correlation results of blinks and eye movements with alternation dynamic.

		Rolling wheel	Vase-face	Binocular rivalry	Necker cube	Rotating cylinder	Moving plaid	Translating diamond
Blink	Pearson *r* value	-0.144	0.067	-0.322	-0.133	0.004	0.133	-0.174
	*p-*value	0.623	0.820	0.261	0.649	0.990	0.651	0.553
Eye movement	Pearson *r* value	-0.060	0.016	0.040	0.133	0.058	-0.063	0.215
	*p*-value	0.837	0.956	0.896	0.651	0.844	0.831	0.460

We also found no correlation between eye movements (measured as standard deviation of distance to center) and the switching rates of the seven types of stimuli (**Table 3**, bottom row). The mean absolute value of *r* is 0.084, and all *p* > 0.45. These results indicate that eye movement mode is also very unlikely the reason for the observed grouping in switching dynamics in the main experiment.

## Discussion

Our results show that the dynamics of different types of bistable phenomena is unlikely controlled by a single mechanism, i.e., there do not seem to be a master mental clock against which all perceptual switches are pegged: a person experiencing faster switching in one phenomenon would not necessarily perceive faster switching in all other bistable stimuli. That said, it is also very apparent from the data that the dynamics of some subsets of bistable stimuli are clearly correlated, forming a number of related groups.

The most noticeable relationships are the correlations among spinning dancer, Lissajous-figure and rotating cylinder. They showed a considerable high correlation to each other. This is not surprising since they are all examples of structure-from-motion stimuli, the bistability of these stimuli from assigning one surface or the other to the front vs. back.

The second group of phenomena includes binocular rivalry, vase-face, moving plaid, Necker cube, and rolling wheel. The factors contributing to this cluster are more complicated. Some in these group of bistable stimuli could be considered having a bistable figure-ground assignments in the general sense, although they differ in which features determine the figure-ground relationship. In the case of binocular rivalry, when one eye’s input becomes the figure, the other recedes into the back and indeed could be considered occluded by the figure. For the Necker cube, the switching between front and back surfaces is more apparent, which is determined by the viewer-cube viewpoint relationship. The vase-face is a clear example of figure-ground bistable assignment, with the figure-ground relationship determined by boarder-ownership. Though Moving Plaid and Rolling Wheel maybe correlated with each other due to shared motion mechanism, it is less clear how these two are related to the other phenomena in this group, though our results also indicated that RW and MP seem to be somewhat removed from the other three (**Figure 6**).

The remaining three bistable phenomena (MB, TD, and BM) seem to be more unique in their underlying neural mechanisms and the key factors determining their bistability. This is particularly true for the point-light Biological Motion (BM) display. Although one could think that BM is also an example of structure-from-motion, numerous studies have suggested that there are dedicated neural mechanisms for the processing of BM, involving cortical regions such as the STS (Grossman et al., 2000; Beauchamp et al., 2003; Grossman et al., 2005).

A number of studies have suggested predictive coding as a possible candidate mechanism for explaining bistable rivalry (Hohwy et al., 2008; Megumi et al., 2015; Weilnhammer et al., 2017; Brascamp et al., 2018). For the hierarchical Bayesian inference, the prediction-error signal generated at each level drives the perceptual switch. The relative independence of some of the switching dynamics in our study may indicate that the prediction-error signals could be generated at different cortical areas for different types of stimuli (e.g., SFM may involve MT+/V5, biological motion may involve STS, and TD may involve LOC).

Gender seems to play an important role in the switching dynamics of the structure-from-motion stimuli (Shechter et al., 1991; Scocchia et al., 2014). Results show that the switching rates in males are significantly slower than that in females for the three structure-from-motion stimuli. However, it is difficult to pinpoint which components of the neural processing are responsible for the observed gender difference.

It is also reported that some of the bistable perception slows down with increasing age (Beer et al., 1989; Ukai et al., 2003; Hudak et al., 2011; Aydin et al., 2013). Subjects in our study have a rather limited age range and the current sample is not the best for observing age-related effects. Still, we did see that the switching rates of translating diamond slowed down with the age. Apparently, the ability to group (and ungroup) individual items to form (and to dissolve) a coherent shape is more flexible in younger adults and deteriorate quite rapidly with increased age, considering the narrow range of our observers’ age.

There were early reports that the switching dynamics of binocular rivalry were correlated with that of the Necker cube (Shannon et al., 2011), motion induced blindness (Carter and Pettigrew, 2003), and stimuli rivalry (Patel et al., 2015). Results from the current study provide further support that binocular rivalry and Necker cube share some common factors in determining their dynamics. However, our data show much lower correlation between binocular rivalry and motion induced blindness compared with what was reported in Carter and Pettigrew. It is not clear whether the differences of stimulus used between the two studies that are responsible for the discrepancy, but subjects in the Carter and Pettigrew’s (2003) study had a wider range of age, making it more likely that age could be the common contributing factor behind the correlation between binocular rivalry and the motion induced blindness, and there is also the possibility that a very small number of subjects with much faster switching dynamics than others accounted for a large portion of the correlation (see their article Figure 1).

Eye movements and eye blinks could potentially play a role in the switching dynamics of bistable perception. One study found a positive correlation between eye movements or blinks with binocular rivalry, but not essential to perceptual switch in other perceptual rivalry (van Dam and van Ee, 2005, 2006b). Their data show that saccades were not associated with perceptual transitions, though blink rate increased around the time of a switch. In the current study, we did observe that subjects had different blink patterns during binocular rivalry sessions compared with other stimuli, potentially due to the use of the stereoscope. A study on Necker cube indicated that changing eye position itself might provide a negative feedback signal that suppresses the percept (Einhäuser et al., 2004). Besides, there were studies reported eye movements have no relationship with rivalry rates, which were consistent with our results (Ee et al., 2003; van Dam and van Ee, 2006a; Law et al., 2015). In our research, individual’s blink rate of each stimulus appeared appropriately constant, which we think shows individual’s blink character. Considering clustered correlation among bistable switching rates, we think there is no much correlation between blink and switch time dynamic. We calculated the eye movement variety, and found no special correlation between eye movement and switch dynamic as well. Overall, the clustering of bistable dynamics among different bistable phenomena is more likely due to the intrinsic mechanisms of those bistable phenomena, rather than the result of different patterns of eye movements or blinks. In addition, the control experiments on the retinal location also provide support that the correlational structure among the different stimuli will be stable across different retina locations, despite that the absolute switching rate could change at different retinal location.

The dynamics of bistable phenomena provide a window into the intrinsic property of temporal operations in the brain, since the perceptual switches were not dictated by changes in the external stimulus, but initiated by the internal processes in the brain. Understanding the nature of bistable dynamics also has important clinical implications. For example, people who suffered from bipolar disorder have a slower switching rates in binocular rivalry and structure-from-motion displays relative to the normal controls (Pettigrew and Miller, 1998; Miller et al., 2003, 2012; Krug et al., 2008; Nagamine et al., 2009; Ngo et al., 2011; Vierck et al., 2013; Law et al., 2015). Our data show that there is not a single “master clock” type mechanism that governs the temporal dynamics of perceptual switches of all bistable stimuli, instead there seem to be a set of different mechanisms responsible for different groups of bistable phenomena. Thus, it is unlikely that patients with mental diseases (e.g., bipolar) would have slower switching for all bistable phenomena, contrary to what was suggested by Miller et al. (2003). Indeed, the potential difference in their relative change in temporal dynamics among patient groups suffering from different types of mental disorders could potentially serve as an objective and effective multi-dimensional endophenotype index in the research and diagnosis of different types of mental disorders.

## Conclusion

We measured the temporal dynamics of 11 bistable phenomena in 100 observers. Results show that the switching rates of subsets of bistable percept are highly correlated, yet different groups of bistable stimuli show relatively independent perceptual switching dynamics. The clustering of dynamic properties of some bistable phenomena but not an overall general control of switching dynamics implies that the temporal pace of bistable perception is not controlled by a “master clock” type mechanism. Instead these results suggest that the existence of both shared and independent inferential processes in the brain responsible for the dynamic of different types of stimuli – faster in constructing 3D structure from motion does not mean quicker in integrating components into an object.

## Ethics Statement

All subjects provided written informed consent before the experiments, and protocols were approved by the institutional review board of the Institute of Biophysics, Chinese Academy of Sciences.

## Author Contributions

SH, TC, SE, and LW contributed to the conception and the design of the work. TC and ZS acquired the data. TC analyzed the data, and wrote the initial draft. SH and SE revised the manuscript.

## Conflict of Interest Statement

The authors declare that the research was conducted in the absence of any commercial or financial relationships that could be construed as a potential conflict of interest.

## References

[B1] Aydin S. Strang N. C. Manahilov V. (2013). Age-related deficits in attentional control of perceptual rivalry. *Vision Res.* 77 32–40. 10.1016/j.visres.2012.11.010 23206550

[B2] Beauchamp M. S. Lee K. E. Haxby J. V. Martin A. (2003). fMRI responses to video and point-light displays of moving humans and manipulable objects. *J. Cogn. Neurosci.* 15 991–1001. 10.1162/089892903770007380 14614810

[B3] Beer J. Beer J. Markley R. P. Camp C. J. (1989). Age and living conditions as related to perceptions of ambiguous figures. *Psychol. Rep.* 64 1027–1033. 10.2466/pr0.1989.64.3c.1027 2762452

[B4] Blake R. Logothetis N. K. (2002). Visual competition. *Nat. Rev. Neurosci.* 3 13–21. 10.1038/nrn701 11823801

[B5] Brascamp J. Sterzer P. Blake R. Knapen T. (2018). Multistable perception and the role of the frontoparietal cortex in perceptual inference. *Annu. Rev. Psychol.* 69 77–103. 10.1146/annurev-psych-010417-085944 28854000

[B6] Brascamp J. W. Blake R. (2012). Inattention abolishes binocular rivalry: perceptual evidence. *Psychol. Sci.* 23 1159–1167. 10.1177/0956797612440100 22933458

[B7] Brascamp J. W. Van Ee R. Pestman W. R. Van Den Berg A. V. (2005). Distributions of alternation rates in various forms of bistable perception. *J. Vis.* 5 287–298. 15929652 10.1167/5.4.1

[B8] Carter O. L. Pettigrew J. D. (2003). A common oscillator for perceptual rivalries? *Perception* 32 295–305. 12729381 10.1068/p3472

[B9] Chong S. C. Blake R. (2006). Exogenous attention and endogenous attention influence initial dominance in binocular rivalry. *Vision Res.* 46 1794–1803. 10.1016/j.visres.2005.10.031 16368126

[B10] Chong S. C. Tadin D. Blake R. (2005). Endogenous attention prolongs dominance durations in binocular rivalry. *J. Vis.* 5 1004–1012. 10.1167/5.11.6 16441198

[B11] Christman S. D. Sontam V. Jasper J. D. (2009). Individual differences in ambiguous-figure perception: degree of handedness and interhemispheric interaction. *Perception* 38 1183–1198. 10.1068/p6131 19817151

[B12] Dieter K. C. Tadin D. (2011). Understanding attentional modulation of binocular rivalry: a framework based on biased competition. *Front. Hum. Neurosci.* 5:155. 10.3389/fnhum.2011.00155 22144958 PMC3228993

[B13] Ee R. Dam L. C. J. Brouwer G. J. Korsten N. J. H. (2003). Bistable stereoscopic 3D percepts: Will-power, flip frequency, eye movements and blinks. *J. Vis.* 3 160–160. 10.1167/3.9.160

[B14] Einhäuser W. Martin K. A. König P. (2004). Are switches in perception of the Necker cube related to eye position? *Eur. J. Neurosci.* 20 2811–2818. 15548224 10.1111/j.1460-9568.2004.03722.x

[B15] Fang F. Kersten D. Murray S. O. (2008). Perceptual grouping and inverse fMRI activity patterns in human visual cortex. *J. Vis.* 8 2–9. 10.1167/8.7.2 19146235

[B16] Grossman E. Donnelly M. Price R. Pickens D. Morgan V. Neighbor G. (2000). Brain areas involved in perception of biological motion. *J. Cogn. Neurosci.* 12 711–720. 10.1162/089892900562417 11054914

[B17] Grossman E. D. Battelli L. Pascual-Leone A. (2005). Repetitive TMS over posterior STS disrupts perception of biological motion. *Vision Res.* 45 2847–2853. 10.1016/j.visres.2005.05.027 16039692

[B18] Helmholtz H. V. (1962). *Treatise on Physiological Optics.* New York, NY: Dover Publications.

[B19] Hohwy J. Roepstorff A. Friston K. (2008). Predictive coding explains binocular rivalry: an epistemological review. *Cognition* 108 687–701. 10.1016/j.cognition.2008.05.010 18649876

[B20] Hudak M. Gervan P. Friedrich B. Pastukhov A. Braun J. Kovacs I. (2011). Increased readiness for adaptation and faster alternation rates under binocular rivalry in children. *Front. Hum. Neurosci.* 5:128. 10.3389/fnhum.2011.00128 22069386 PMC3208241

[B21] Kanai R. Bahrami B. Rees G. (2010). Human parietal cortex structure predicts individual differences in perceptual rivalry. *Curr. Biol.* 20 1626–1630. 10.1016/j.cub.2010.07.027 20727757 PMC2949566

[B22] Kanai R. Carmel D. Bahrami B. Rees G. (2011). Structural and functional fractionation of right superior parietal cortex in bistable perception. *Curr. Biol.* 21 R106–R107. 10.1016/j.cub.2010.12.009 21300270 PMC3084447

[B23] Kanai R. Moradi F. Shimojo S. Verstraten F. A. (2005). Perceptual alternation induced by visual transients. *Perception* 34 803–822. 10.1068/p5245 16124267

[B24] Klink P. C. Van Ee R. Nijs M. M. Brouwer G. J. Noest A. J. Van Wezel R. J. A. (2008). Early interactions between neuronal adaptation and voluntary control determine perceptual choices in bistable vision. *J. Vis.* 8 16.1–18. 10.1167/8.5.16 18842087

[B25] Knapen T. Brascamp J. Pearson J. Van Ee R. Blake R. (2011). The role of frontal and parietal brain areas in bistable perception. *J. Neurosci.* 31 10293–10301. 10.1523/JNEUROSCI.1727-11.2011 21753006 PMC3146344

[B26] Kohler A. Haddad L. Singer W. Muckli L. (2008). Deciding what to see: the role of intention and attention in the perception of apparent motion. *Vision Res.* 48 1096–1106. 10.1016/j.visres.2007.11.020 18279907

[B27] Krug K. Brunskill E. Scarna A. Goodwin G. M. Parker A. J. (2008). Perceptual switch rates with ambiguous structure-from-motion figures in bipolar disorder. *Proc. R. Soc. Lond. B Biol. Sci.* 275 1839–1848. 10.1098/rspb.2008.0043 18463054 PMC2494571

[B28] Law P. C. F. Paton B. K. Riddiford J. A. Gurvich C. T. Ngo T. T. Miller S. M. (2015). No relationship between binocular rivalry rate and eye-movement profiles in healthy individuals: a bayes factor analysis. *Perception* 44 643–661. 10.1177/0301006615594267 26489208

[B29] Lehky S. R. (1995). Binocular rivalry is not chaotic. *Proc. R. Soc. Lond. B Biol. Sci.* 259 71–76. 10.1098/rspb.1995.0011 7700878

[B30] Leopold D. A. Logothetis N. K. (1999). Multistable phenomena: changing views in perception. *Trends Cogn. Sci.* 3 254–264. 10.1016/S1364-6613(99)01332-7 10377540

[B31] Liu C.-H. Tzeng O. J. Hung D. L. Tseng P. Juan C.-H. (2012). Investigation of bistable perception with the “silhouette spinner”: Sit still, spin the dancer with your will. *Vision Res.* 60 34–39. 10.1016/j.visres.2012.03.005 22465540

[B32] Lumer E. D. Friston K. J. Rees G. (1998). Neural correlates of perceptual rivalry in the human brain. *Science* 280 1930–1934. 10.1126/science.280.5371.1930 9632390

[B33] Megumi F. Bahrami B. Kanai R. Rees G. (2015). Brain activity dynamics in human parietal regions during spontaneous switches in bistable perception. *Neuroimage* 107 190–197. 10.1016/j.neuroimage.2014.12.018 25512040 PMC4306523

[B34] Meng M. Tong F. (2004). Can attention selectively bias bistable perception? Differences between binocular rivalry and ambiguous figures. *J. Vis.* 4 539–551. 10.1167/4.7.2 15330700 PMC1403736

[B35] Miller S. Gynther B. Heslop K. Liu G. Mitchell P. Ngo T. (2003). Slow binocular rivalry in bipolar disorder. *Psychol. Med.* 33 683–692. 10.1017/S0033291703007475 12785470

[B36] Miller S. Ngo T. Van Swinderen B. (2012). Attentional switching in humans and flies: rivalry in large and miniature brains. *Front. Hum. Neurosci.* 5:188. 10.3389/fnhum.2011.00188 22279432 PMC3260559

[B37] Miller S. M. Liu G. B. Ngo T. T. Hooper G. Riek S. Carson R. G. (2000). Interhemispheric switching mediates perceptual rivalry. *Curr. Biol.* 10 383–392. 10.1016/S0960-9822(00)00416-4 10753744

[B38] Nagamine M. Yoshino A. Miyazaki M. Takahashi Y. Nomura S. (2009). Difference in binocular rivalry rate between patients with bipolar I and bipolar II disorders. *Bipolar Disord.* 11 539–546. 10.1111/j.1399-5618.2009.00719.x 19624393

[B39] Nagamine M. Yoshino A. Yamazaki M. Obara M. Sato S.-I. Takahashi Y. (2007). Accelerated binocular rivalry with anxious personality. *Physiol. Behav.* 91 161–165. 10.1016/j.physbeh.2007.02.016 17433385

[B40] Ngo T. T. Mitchell P. B. Martin N. G. Miller S. M. (2011). Psychiatric and genetic studies of binocular rivalry: an endophenotype for bipolar disorder? *Acta Neuropsychiatr.* 23 37–42. 10.1111/j.1601-5215.2010.00510.x

[B41] Pastukhov A. Braun J. (2011). Cumulative history quantifies the role of neural adaptation in multistable perception. *J. Vis.* 11:12. 10.1167/11.10.12 21931128

[B42] Patel V. Stuit S. Blake R. (2015). Individual differences in the temporal dynamics of binocular rivalry and stimulus rivalry. *Psychon. Bull. Rev.* 22 476–482. 10.3758/s13423-014-0695-1 25092387 PMC4318784

[B43] Pearson J. Brascamp J. (2008). Sensory memory for ambiguous vision. *Trends Cogn. Sci.* 12 334–341. 10.1016/j.tics.2008.05.006 18684661

[B44] Pettigrew J. D. Miller S. M. (1998). A ‘sticky’ interhemispheric switch in bipolar disorder? *Proc. R. Soc. B Biol. Sci.* 265 2141–2148. 10.1098/rspb.1998.0551 9872002 PMC1689515

[B45] Schouten B. Davila A. Verfaillie K. (2013). Further explorations of the facing bias in biological motion perception: perspective cues, observer sex, and response times. *PLoS One* 8:e56978. 10.1371/journal.pone.0056978 23468898 PMC3584127

[B46] Schouten B. Troje N. E. Brooks A. Van Der Zwan R. Verfaillie K. (2010). The facing bias in biological motion perception: effects of stimulus gender and observer sex. *Attent. Percept. Psychophys.* 72 1256–1260. 10.3758/APP.72.5.1256 20601707

[B47] Scocchia L. Valsecchi M. Triesch J. (2014). Top-down influences on ambiguous perception: the role of stable and transient states of the observer. *Front. Hum. Neurosci.* 8:979. 10.3389/fnhum.2014.00979 25538601 PMC4259127

[B48] Shannon R. W. Patrick C. J. Jiang Y. Bernat E. He S. (2011). Genes contribute to the switching dynamics of bistable perception. *J. Vis.* 11:8. 10.1167/11.3.8 21389101 PMC3962018

[B49] Shechter S. Hillman P. Hochstein S. Shapley R. M. (1991). Gender differences in apparent motion perception. *Perception* 20 307–314. 10.1068/p200307 1762873

[B50] Sheppard B. M. Pettigrew J. D. (2006). Plaid motion rivalry: correlates with binocular rivalry and positive mood state. *Perception* 35 157–169. 10.1068/p5395 16583762

[B51] Shi J. Weng X. He S. Jiang Y. (2010). Biological motion cues trigger reflexive attentional orienting. *Cognition* 117 348–354. 10.1016/j.cognition.2010.09.001 20883983 PMC2967601

[B52] Sterzer P. Kleinschmidt A. (2007). A neural basis for inference in perceptual ambiguity. *Proc. Natl. Acad. Sci. U.S.A.* 104 323–328. 10.1073/pnas.0609006104 17190824 PMC1765459

[B53] Sterzer P. Kleinschmidt A. Rees G. (2009). The neural bases of multistable perception. *Trends Cogn. Sci.* 13 310–318. 10.1016/j.tics.2009.04.006 19540794

[B54] Ukai K. Ando H. Kuze J. (2003). Binocular rivalry alternation rate declines with age. *Percept. Mot. Skills* 97 393–397. 10.2466/pms.2003.97.2.393 14620224

[B55] van Dam L. C. van Ee R. (2005). The role of (micro) saccades and blinks in perceptual bi-stability from slant rivalry. *Vision Res.* 45 2417–2435. 10.1016/j.visres.2005.03.013 15894347

[B56] van Dam L. C. J. van Ee R. (2006a). Retinal image shifts, but not eye movements per se, cause alternations in awareness during binocular rivalry. *J. Vis.* 6 1172–1179. 10.1167/6.11.3 17209727

[B57] Van Dam L. C. van Ee R. (2006b). The role of saccades in exerting voluntary control in perceptual and binocular rivalry. *Vision Res.* 46 787–799. 16309727 10.1016/j.visres.2005.10.011

[B58] van Ee R. (2005). Dynamics of perceptual bi-stability for stereoscopic slant rivalry and a comparison with grating, house-face, and Necker cube rivalry. *Vision Res.* 45 29–40. 10.1016/j.visres.2004.07.039 15571736

[B59] van Ee R. (2009). Stochastic variations in sensory awareness are driven by noisy neuronal adaptation: evidence from serial correlations in perceptual bistability. *J. Opt. Soc. Am. A Opt. Image Sci. Vis.* 26 2612–2622. 10.1364/JOSAA.26.002612 19956332

[B60] Vierck E. Porter R. J. Luty S. E. Moor S. Crowe M. T. Carter J. D. (2013). Further evidence for slow binocular rivalry rate as a trait marker for bipolar disorder. *Aust. N. Z. J. Psychiatry* 47 371–379. 10.1177/0004867412474105 23341474

[B61] Weilnhammer V. Ludwig K. Sterzer P. Hesselmann G. (2014). Revisiting the Lissajous figure as a tool to study bistable perception. *Vision Res.* 98 107–112. 10.1016/j.visres.2014.03.013 24718018

[B62] Weilnhammer V. Stuke H. Hesselmann G. Sterzer P. Schmack K. (2017). A predictive coding account of bistable perception-a model-based fMRI study. *PLoS Comput. Biol.* 13:e1005536. 10.1371/journal.pcbi.1005536 28505152 PMC5448813

[B63] Weilnhammer V. A. Ludwig K. Hesselmann G. Sterzer P. (2013). Frontoparietal cortex mediates perceptual transitions in bistable perception. *J. Neurosci.* 33 16009–16015. 10.1523/JNEUROSCI.1418-13.2013 24089505 PMC6618467

[B64] Windmann S. Wehrmann M. Calabrese P. Gunturkun O. (2006). Role of the prefrontal cortex in attentional control over bistable vision. *J. Cogn. Neurosci.* 18 456–471. 10.1162/jocn.2006.18.3.456 16513009

[B65] Zhang P. Jamison K. Engel S. He B. He S. (2011). Binocular rivalry requires visual attention. *Neuron* 71 362–369. 10.1016/j.neuron.2011.05.035 21791293 PMC3175243

